# Eating and Drinking Recognition in Free-Living Conditions for Triggering Smart Reminders

**DOI:** 10.3390/s19122803

**Published:** 2019-06-22

**Authors:** Diana Gomes, João Mendes-Moreira, Inês Sousa, Joana Silva

**Affiliations:** 1 Fraunhofer Portugal AICOS, 4200-135 Porto, Portugal; ines.sousa@fraunhofer.pt (I.S.); joana.silva@fraunhofer.pt (J.S.); 2 LIAAD-INESC TEC, 4200-465 Porto, Portugal; joao.mendes.moreira@inesctec.pt; 3 Faculty of Engineering, University of Porto, 4200-465 Porto, Portugal

**Keywords:** classification, data segmentation, drinking recognition, eating recognition, elderly care, human activity recognition

## Abstract

The increasingly aging society in developed countries has raised attention to the role of technology in seniors’ lives, namely concerning isolation-related issues. Independent seniors that live alone frequently neglect meals, hydration and proper medication-taking behavior. This work aims at eating and drinking recognition in free-living conditions for triggering smart reminders to autonomously living seniors, keeping system design considerations, namely usability and senior-acceptance criteria, in the loop. To that end, we conceived a new dataset featuring accelerometer and gyroscope wrist data to conduct the experiments. We assessed the performance of a single multi-class classification model when compared against several binary classification models, one for each activity of interest (eating vs. non-eating; drinking vs. non-drinking). Binary classification models performed consistently better for all tested classifiers (*k*-NN, Naive Bayes, Decision Tree, Multilayer Perceptron, Random Forests, HMM). This evidence supported the proposal of a semi-hierarchical activity recognition algorithm that enabled the implementation of two distinct data stream segmentation techniques, the customization of the classification models of each activity of interest and the establishment of a set of restrictions to apply on top of the classification output, based on daily evidence. An F1-score of 97% was finally attained for the simultaneous recognition of eating and drinking in an all-day acquisition from one young user, and 93% in a test set with 31 h of data from 5 different unseen users, 2 of which were seniors. These results were deemed very promising towards solving the problem of food and fluids intake monitoring with practical systems which shall maximize user-acceptance.

## 1. Introduction

Society’s aging brings many problems. The growth of the average life expectancy is due to the evolution of health systems, the pharmacological advances and the availability of biocompatible devices, associated with overall improved life conditions. However, costs with health remain on the table, with ethical and political considerations always in the loop. The seniors scenario is also worrisome due to isolation-related issues. More often than not, seniors do not want to abandon their homes or lose their independence and hiring assistance from a professional can be very expensive. For that reason, the older adults frequently neglect essential daily activities, like eating properly, hydrating or taking care of their personal hygiene [1]. The spread of smartphones has opened new windows and cleared room for improvements even in the health sector. Wearable devices, like smartwatches, are also recognized for their high potential and utility to monitor activities (e.g., physical exercise). This technological context has been providing a basis for the development of effective and low-cost health solutions, which are currently in high demand.

The relation food–water–medication is very worrisome in the older adults. There are, in fact, several medicines associated to the current status of the digestive system (must be taken with empty stomach, during or after a meal). By forgetting or deliberately skipping a meal, one will probably forget medication or improperly ensure the treatment. Therefore, in this context, it is very interesting to be able to detect when a user performs a meal or drinks a glass of water, since it would enable proper monitoring by the care-providers and can even be the basis for the issuing of automatic reminders in the most opportune situations along the day. This relies on the ability to correctly identify eating and drinking moments in real-time and real-world conditions. Moreover, establishing seniors as the target population of a technological solution means that simplicity and unobtrusiveness of the system are important prerequisites.

This work aims to address this need by proposing a new user-independent algorithm for simultaneous eating and drinking recognition in real-time and free-living conditions. The system should be unobtrusive and practical, so it does not disturb seniors’ activities and is easily accepted by them. Therefore, it was based on wrist sensorization, following the considerations of several works on the acceptance of wrist-worn devices by older adults for activity monitoring purposes [2,3,4]. Moreover, features of low computational cost [5] and/or which have been associated with appropriate reported results in Human Activity Recognition (HAR) works, namely those which recognize the activities of interest, were preferred and implemented, aiming the feasibility and efficiency of an eventual future smartphone application implementing the proposed algorithm. Validation took place following a two-stage approach which assessed its potential for all-day utilization along with its user-independent nature, achieving F1-scores of 97% and 93%, respectively.

Several studies were surveyed in the scope of this study, either for comparison of approaches [6,7,8,9,10] or to explore the proposed state-of-the-art techniques framed within our work’s constraints and objectives [5,7,11,12,13]. Among the main contributions of this work, we can highlight the following:

- Introduction of a new dataset to assist the creation of eating and drinking recognition algorithms, focused on capturing the diversity of wrist/hand movements inherent to free-living conditions utilization;
- Assessment of the impact of eating and drinking simultaneous recognition through a semi-hierarchical approach, based on two binary classifiers rather than a single multi-class classifier;
- Assessment of the impact of adding several eating and drinking-related restrictions in a post-processing layer, acting on top of the classification output;
- Proposal of a new application for the algorithm of dynamic segmentation of data streams proposed by [11], and a variation of that same algorithm which improved drinking recognition sensitivity when compared to the standard approach of that work.

This document is divided into six main sections. First, an introduction to the context, problem and motivation is presented, followed by a review of what was already accomplished towards eating and drinking recognition. Section 3 clarifies the methods behind the proposed algorithm, and is followed by the presentation of the results of the experiments. A thorough discussion of results is promoted in Section 5, and the manuscript is closed by a final section drawing the main conclusions and pointing out future work.

## 2. Background

Human Activity Recognition (HAR) is a recent and very broad area. In particular, the recognition of Activities of Daily Living (ADL) has been extensively studied, using different data sources (inertial, image, RFID) and techniques. However, for application in free-living, monitoring systems must be overall appealing, so that users adhere to them. Inertial data seems to be associated to fewer privacy concerns than the acquisition and processing of image data, even though computer vision techniques have been popular for ADL recognition [14].

The related work overview of this section has, thus, especial focus in inertial data-based approaches, following several techniques of time series analysis, further discussed and framed within the purpose of this work.

### 2.1. Distinguishing Eating and Drinking Activities from other ADL

There are HAR systems that specifically focus on ADL recognition. While many of these works are confined to the study of ambulatory activities, it is possible to find a few that recognize eating. Table 1 summarizes some of these works.

By aiming to recognize complex activities (such as the activities of interest), which not all HAR works focus on, the works of Table 1 faced particular challenges and drawn interesting conclusions. Shoaib et al. studied the influence of increasingly larger windows (2 to 30 s) and concluded that a larger window size is associated with better performance in recognizing complex activities [12]. As it is not possible to increase the window size indefinitely, the authors recommend the implementation of an hierarchical approach (on top of the classification output) in case of activities that inherently include pauses among gestures or other unpredictable movements, as eating. Hierarchical activity recognition algorithms were also explored in [1], allied to reduced computational cost. As far as we know, the work of Shoiab et al. is also the only work that simultaneously recognizes both eating and drinking [12] (the authors in [15] also recognize both activities, but do not distinguish one from the other). It is also interesting to acknowledge the work of Chernbumroong et al. for focusing in the same target population as the present work, older adults [17], and that of Zhu, which distinguishes 10 high-level daily activities simply resorting to 2 features and a Dynamic Bayesian Network [16].

Very few works seem to distinguish the drinking activity from other ADL. Notwithstanding, the work of [12] reports up to 91% F-measure in recognizing drinking. In [18], the authors also distinguish eating and drinking from smoking, writing, typing and brushing the teeth in an experiment to assess their algorithm performance in distinguishing similar activities. Even though it was a small experiment, with only one test subject, an overall accuracy of roughly 98% was attained.

This context may be an evidence of the challenge of optimizing the user-acceptance/performance trade-off when developing solutions for simultaneous eating and drinking recognition and distinction. This is a consequence of several technical challenges. First, there is the problem of handling class unbalance, since, in free-living, both activities are sporadic and rare, and drinking, in particular, is typically of very short duration. Both activities also involve handling tools (e.g., cutlery, cups) and the hand-to-mouth gesture, movements which can be monitored with hand/wrist sensorization (typically easily accepted by seniors). Collecting wrist data to make such predictions is, thus, both adequate and challenging, due to the possibility of confusion of the activities of interest through the sole use of gesture spotting approaches. As such, these activities are frequently gathered as a whole in a single “eat/drink” class, which would not suit our purpose within this work. This evidences the particular challenges of addressing eating and drinking identification under the constraints of our unobtrusive system design, with additional requirements of near real-time response, free-living conditions, and user-independence, with appropriate performance.

### 2.2. Eating Recognition

The surveyed works confirm the assumption that inertial data would be an appropriate approach in recognizing the eating activity. Nevertheless, Thomaz has proposed several ways of detecting eating moments, taking advantage of different types of sensor data to do so [19]. One of the approaches uses first-person point of view photographs and computer vision techniques allied with Convolutional Neural Networks (CNN) classification. Even though the main focus of the work was eating detection, it recognized 19 ADL from one individual over a period of 6 months. It was possible to achieve a total accuracy for all 19 activities, and the eating activity itself, of roughly 83%. The author also conducted an experiment with audio signals, which resulted in eating activities being identified with 90% precision and 76% recall.

Several works focused in gesture detection to predict eating periods [6,13,20,21,22], proposing segmentation methods of said gestures in the continuous data stream and their classification. However, it is important to acknowledge the heavy and time-consuming labeling job, especially for large datasets, as the aforementioned works remarkably present. Moreover, the subjectivity of this process is very worrisome, as different people might not agree in the beginning and ending moments of an activity and/or gesture. Gesture spotting approaches often resort to classifiers that instigate sequential dependency, since gestures are frequently performed sequentially. This is why Hidden Markov Models (HMM) are one of the most employed classification methods, namely when dealing with continuous data streams from motion sensors. In [20], the authors discriminated eating an drinking gestures from other movements with an accuracy of 95% on isolated gesture segments and 87% with online streaming data. Later on, a study of recognition of swallowing, chewing and arm movements (e.g., hand-to-mouth) food intake events was performed in a multi-modal approach with resource to 7 sensors (inertial, EMG and audio) [21]. Some works proposed an explicit segmentation as a preprocessing step to improve the spotting task [23,24], instead of relying on HMMs intrinsic segmentation capabilities. Believing in the importance of that step, a two-staged spotting approach was proposed, firstly identifying potential candidate sections in the data stream and only then proceeding to classification with HMMs in order to remove false positives (non-relevant gestures) [22]. In this work, it was also presented a case study that aimed to distinguish between eating with cutlery, drinking, eating with spoon and eating with hands, with an overall recall and precision of 79% and 73%, respectively. Ramos-Garcia et al. also tracked wrist motion with inertial sensors [13]. Following the work of [7], where they performed eating detection continuously throughout an entire day with simple, while effective, algorithms, hierarchical HMMs that take advantage of the gesture-to-gesture sequential dependency were conceived. Their method improved the recognition of eating gestures, achieving finally an overall accuracy of 97%.

The aforementioned works process data acquired in controlled environments rather than free-living conditions. On the contrary, Table 2 summarizes some eating recognition works that validated their algorithms with datasets acquired in free-living conditions. These works aim, however, especially dietary monitoring and food journaling rather than the issuing reminders of any kind, which appears to be a novel intention. Dong et al. highlighted that, in the course of a typical day, the ratio between eating and performing other activities is 1:20 [7]. Therefore, the authors assessed the performance of their method by computing weighted accuracy, balancing true positive and negative predictions’ rate. In the same work, the group verifies that an eating moment is preceded and succeeded by a peak of the energy of the accelerometer’s signal, due to the fact that eating corresponds to a period of essentially small movements which present themselves as a “valley” in the energy signal. This approach, however, lacks real-time response. Thomaz introduced an eating moment estimation approach based in detecting food intake gestures [6], but HMM were not employed. It was possible to recognize eating moments with F1-scores of 76%, for data acquired by 7 participants during 1 day, and 71% for 1 participant over 31 days. Best results of classification of eating gestures was achieved with the Random Forest classifier and eating moments were estimated using the DBSCAN clustering algorithm. The same algorithm was used in [19] with inertial data acquired from Google Glass, and an F1-score of 72% was obtained. In a final experiment, the author used inertial data from both wrists. The results of this experiment were worse than the previous, using the same algorithm. The works reported in [8,9] appear to present the best results. Nevertheless, their systems are more obtrusive, requiring sensors in several locations, while [7] and [6] only use data from one wrist. Their methods also rely in the extraction of a high number of features, some of which from the frequency domain, increasing computational cost.

### 2.3. Drinking Recognition

Drinking recognition is also an interesting task. In fact, in [10], the authors go even further and aim to determine fluid intake. They achieved 84% recall and 94% precision in recognizing drinking motion from a continuous data stream, with only one inertial wrist sensor. Then, container types and fluid level were estimated. Even though all experiments took place in a controlled environment, a suggestive 75% and 72% recognition rate of container type and fluid level, respectively, was achieved with a C4.5 Decision Tree classifier. The same authors had already tried to distinguish drinking from eating movements [20]. However, this work focused solely in distinguishing gestures, i.e., it did not aim to recognize drinking (and eating) among other activities performed in the course of a day.

Recently, a new approach for the problem of drinking recognition with inertial sensors was proposed in [25]. The authors detected the hand-to-mouth movement before the a sipping event with a F1-score of 97% for their offline validation and 85% for the real-time validation.

## 3. Methodology

This section describes the experiments conducted in the scope of this work. Their implementation was performed in Python language, using some well-know toolboxes, namely scikit-learn [26].

### 3.1. Dataset Acquisition

The general purpose of eating and drinking recognition in daily living conditions required a dataset that featured data collected in free-living conditions, with at least three well-represented classes—eat, drink and other, which includes all non-eating and drinking activities, from a reasonable number of volunteers. Due to the complexity of the activities, it was necessary to guarantee that the diversity of movements involved in performing both activities of interest was captured. Since no publicly available dataset was found to address this set of needs, a data acquisition step took place.

Wrist inertial data was acquired using a wearable sensing device, called *Pandlets*, and a smartphone. Pandlets were developed by Fraunhofer Portugal AICOS, designed for ultra-low power consumption, to simplify integration with mobile platforms and to respond to developers’ needs [27]. These are composed by the Pandlets CORE, which features inertial and environmental sensors. The *Pandlets Recorder* application was used to connect the devices to a smartphone, and record data from the accelerometer and gyroscope sensors at 100 Hz for every acquisition. *Pandlets* were placed in a watch-like conformation in the dominant wrist or both wrists (depending on the protocol), held with a strap. The orientation of the sensors was roughly the same for every acquisition and user, since it is a common daily practice to place wrist accessories (e.g., watches, bracelets) with similar orientation every time. In order to address all the objectives of acquisition, a total of three datasets was attained (Table 3).

#### 3.1.1. Dataset 1: Perfectly Segmented Activities

Dataset 1 was planned to enable the understanding of the differences between the signals of the activities of interest and other daily living activities. Therefore, it was composed of well-segmented activities, featuring the three classes required. Since the activities of interest are complex, several ways of performing them were specified in order to originate a complete, heterogeneous and general dataset. In this sense, the acquisition protocol included describing each activity to the volunteers before they were asked to execute it (see Table 3), without placing any movements’ restriction. This dataset accounted for a total of 11 volunteers, with ages ranging from 18 to 65 years old. Some users were not able to perform all activities due to time constraints. Data from the accelerometer and gyroscope placed on both wrists was recorded.

#### 3.1.2. Dataset 2: Continuous Sequence of Activities

In order to capture the diversity of eating gestures involved in a meal, drinking in different contexts, transitions between activities and movements as natural as possible, another dataset was acquired. Dataset 2 was conceived by asking 5 volunteers to perform a certain sequence of activities, while inertial data was continuously recorded. It was also asked the explicit consent of the volunteers for video recording of the performed activities, which provided ground truth for data annotation. The sequence of activities was well-defined and explained to the volunteers, with ages ranging from 21 to 65 years-old, so that diversity was guaranteed and a few key activities were performed and recorded, such as eating with different tools and drinking from different containers in several contexts. Data from accelerometer and gyroscope were collected from both wrists of the users. Volunteers were asked to raise both arms at the same time, in a fast movement, producing an isolated acceleration peak, easily detected with both accelerometers. These peaks, detected in both sensing devices, were assumed to correspond to the same moment and used to correct potential time offset between sensors, and enable synchronization with the video. Kinovea [28], an open source software for video analysis that allows the input of comments with an associated time-stamp, was used to assist this time-consuming process. The same tool also allowed the identification of sensor synchronization peaks with frame-precision. Three classes were identified: eat, drink, other.

#### 3.1.3. Dataset 3: Eating and Drinking by Seniors

The need for another dataset was brought forward to increase the robustness and comprehensiveness of the model. Shen et al. acknowledged that age influenced eating recognition by roughly 4% in accuracy [29]. Therefore, when developing a tool suited for seniors, it should also be beneficial to include data acquired from the target population in the training set. That was the purpose of Dataset 3.

This dataset was attained later on in the process, at a time when some decisions had already been made. Thus, it only comprises inertial data from the dominant hand of the user. Moreover, it focuses on longer eating periods, capturing the diversity of movements older adults are expected to perform in the course of a meal. Eating data was acquired from a total of 10 individuals, 6 of which were having lunch, while 4 were having an afternoon snack. Additionally, 18 seniors were asked to deliberately perform the drinking movement to enhance the diversity of drinking data in the training set. However, some recordings had to be excluded from the final dataset due to a loss of inertial sensors’ data during the recording period. Out of the total of volunteers, there were 14 females and 4 males, all of which consented the participation in data collection. Due to privacy concerns, the activities were not video recorded: eating data collection began approximately 1 min into the starting of a meal and stopped slightly before its end, while drinking simulations were recorded from the moment the users were ready to go from a resting position to grabbing a fluid container until the moment they put the container back down.

#### 3.1.4. Validation Sets

Data from 5 different subjects was collected to constitute an in-the-wild validation set with a total of 31 h of data, following 2 distinct protocols.

Firstly, a new unseen dataset was assembled with data from 4 users, two of which were seniors (79 and 86 years old) from the day care center previously introduced, which did not participate in the data collection of the training set. Due to sensor limitations and the day care center’s daily dynamic, we were only able to collect ≈3 h of data for one individual and ≈2 h for the other, including a lunch period. During that time, both volunteers drank water 1 time each. The two remaining subjects were young (22 and 23 years old), and data was continuously recorded over 5 h each, including lunch periods and a total of 29 sips of water. The users kept a manual log of activities, except for the seniors, for whom starting and ending times of activities were annotated by a third person. This protocol was preferred to video recording as ground truth, due to privacy issues, especially in the day care center, where the acquisitions with the seniors took place. Users were asked to perform every activity as they would in a typical day, disregarding the sensor placed in the dominant wrist.

Then, data from another young subject (21 years old) was collected over a 16 h period (from morning till late in the evening), excluding the 8 h of sleep. This user also kept a manual log of activities, annotating starting and ending times of eating and drinking activities with second precision and providing a summary of the other performed activities per hour, which constituted the ground truth. Analogously to the previous test set acquisition protocol, this method was preferred to video recording of the activities to disregard privacy concerns, since some of the user’s activities took place outdoors and assumed direct contact with other people. During this time, the user performed 3 meals—breakfast (8:30 a.m.), lunch (12 a.m.) and dinner (7:30 p.m.)—and drank something on 34 occasions. Eating periods corresponded to 3.8% of the total time, and drinking to 0.7%. The performed meals included moments when the user ate with fork and knife, spoon and hands. The utensil used for drinking also varied (cup, mug and bottle).

### 3.2. Data Preprocessing and Segmentation

Data was down-sampled by a factor of 2 (i.e., to 50 Hz), in order to optimize the trade-off between data acquisition and battery life of the devices involved in the future. The preprocessing steps involved low-pass Butterworth filtering of the data followed by Gaussian smoothing. In order to enable its online usage, only half of a Gaussian distribution was used [7].

**Figure 1:**
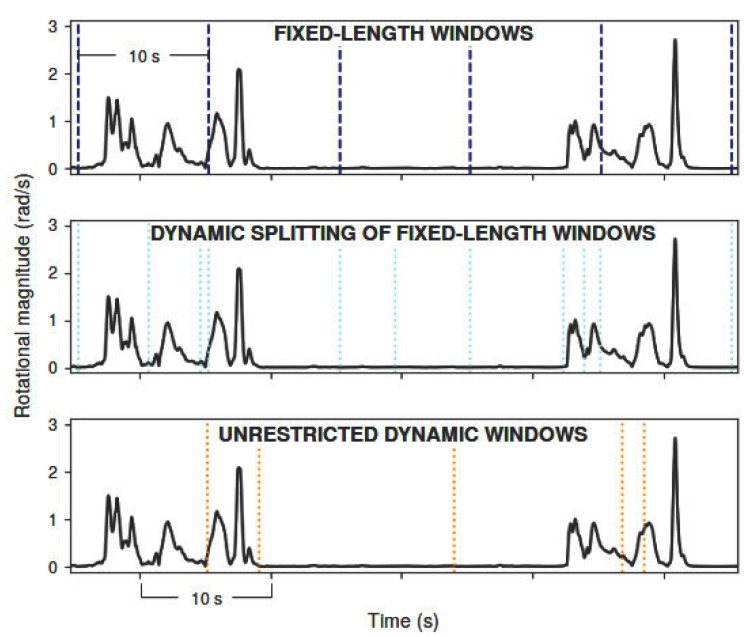
Segmentation results of a drinking activity (2 sips) following different windowing approaches. Vertical lines divide segmented windows. Unrestricted dynamic windows correspond to the direct implementation of the algorithm of [11].Segmentation results of a drinking activity following different windowing approaches.

Eating and drinking are activities with very different average duration. While drinking takes only a few seconds (usually less than 10 s), the eating activity often takes several min. In this sense, three distinct windowing methods were explored (Figure 1).

The first method consisted in traditional fixed-length windows (FW), in which data is added to the window until its length meets the requirement. These windows can overlap in a certain percentage, meaning that some samples would belong to at least two consecutive windows.

In [11], a new online dynamic signal segmentation approach (DW) for data streams was proposed. The authors compared it with the typical fixed-length windows (both overlapping and non-overlapping) reporting better accuracy for their method in the classification of ambulatory and transitional activities. This method is attractive for the present challenge since the activities of interest are expected to have very different duration and, so, fixed-length windows may not be an adequate solution. It splits the data whenever a significant change is detected in the data stream. This significant change corresponds to a descending sequence in which the difference between the maximum and minimum value is larger than a dynamic threshold, computed from the previous *N* data samples. The authors also alert for the importance of selecting an adequate input signal to base threshold computation, as the variation of this signal should be representative of the activities of interest in order for the algorithm to perform well. In other words, it is important to select a meaningful signal from which to compute the parameters and split the data stream. For our application, best results were achieved using the magnitude of the gyroscope signal as the input. The *N* parameter was set to 100 previous samples (2 s).

In this work, we propose a variation of the segmentation approach of [11], in which threshold computation should be reset every *t* s, while data is automatically split at that moment. Since the dynamic segmentation of Kozina et al. [11] relies on a threshold computed at each new sample from the *N* previous samples, window-splitting depends on each new sample’s before-context, which may lead to more unpredictable windows. This might impair drinking activity segmentation in particular, due to its very short duration. With our approach, even varying the previous activity, it is certain that at each *t* seconds there is an opportunity of disregarding past data, and constituting more coherent windows. Moreover, it restrains the maximum length of dynamic windows to *t* seconds. This approach can be summarized as a dynamic splitting of fixed-length windows (DFW).

### 3.3. Feature Extraction and Selection

Ten time-domain features were implemented: mean, standard deviation, variance, axis covariance, axis correlation, Haar-like features [30], manipulation [7,13], linear acceleration [7,13], zero-crossings rate, root mean square. The criteria for the selection of this feature set was low computational load [5], which is important if we consider running the algorithm in a smartphone, and good reported results in HAR works, in particular those which recognize the activities of interest. Manipulation and linear acceleration were implemented as in [7,13]. Mean, standard deviation, variance, zero-crossings rate and Haar-like features were computed for the signal of each axis of both accelerometer and gyroscope. Axis covariances and correlations were calculated by combining the 3-axis of each sensor pairwise. Root mean square was computed from the magnitude of the accelerometer and gyroscope.

In a first step of feature selection, highly correlated features were eliminated, i.e., the most computationally inefficient feature of a feature pair with absolute correlation larger than 0.8 was eliminated. Then, we took advantage of Random Forest’s inherent capabilities for feature ranking based on its computation of Gini importance [31], by training two distinct Random Forest classifiers with features computed from Dataset 1: the first was fed eating vs. non-eating labels, and the second drinking vs. non-drinking labels. Features which verified less than 1% importance in this ranking (for at least one of the activities of interest) were eliminated from the final feature set. The feature selection step resulted, finally, in a vector of length 33, with mean, correlation, variance and Haar-like features computed from all axes of both accelerometer and gyroscope, along with the covariance of accelerometer’s axis, zero-crossings rate of the gyroscope’s axis, manipulation, linear acceleration and root mean square of the magnitude of the accelerometer signal.

### 3.4. Class Balancing Methods and Classification

Dataset 1 was the only one in which activities were planned and defined with time restrictions, i.e., each activity was associated with a duration, which resulted in the only class-balanced dataset. Assembling data from datasets 1 to 3, eating was the most well-represented class, with a total of approximately 5 h of data, and drinking the rarest (approximately 1 h 10 min), due to the nature of the movement itself. Class other comprised a total of 3 h of data relative to all non-eating and drinking activities. This implied that a class balancing strategy should be implemented before the classification stage. *SMOTE + ENN* combines the Synthetic Minority Over-sampling TEchnique (SMOTE) [32] and the Edited Nearest Neighbor (ENN) [33] algorithms, with very good reported results [34,35]. This approach was selected and applied before training each classifier, using the imbalanced-learn package implementation, which is compatible with scikit-learn.

The classification step took place comparing the performance of 6 different supervised classifiers: *k*-NN, Naive Bayes (NB), Decision Tree (DT), HMM, MLP, and Random Forests. HMM-based classification took place by training three different models: the first, only with eating data (5 states), the second only with drinking data (2 states), and the third with data from class other of the acquired datasets (7 states). The number of states parameter was tuned based on previous eating activity-related works [13] and empirical evidence. Whenever a new sequence of observations was to be classified, it passed through all three models and was assigned the label corresponding to the most likely predicted state sequence.

### 3.5. Experiments Description

The description of all performed experiments is subsequently presented. All tests were conducted to assess the performance of the algorithm while preserving user-independence, i.e., all validations took place either by leave-one-subject-out validation (if using the same dataset for training and testing) or by using a testing set which did not feature data from users of the training set.

#### 3.5.1. Sensor Selection

As first step, we intended to determine whether to process data solely from the dominant wrist or both wrists. Moreover, it was important to study if we should collect data from both accelerometer and gyroscope or if one of these sensors would suffice.

In this sense, Dataset 1 was labeled in three classes—eat, drink, other—and segmented in windows of 10 s with 25% overlap, from which two features were extracted (mean and standard deviation). Features were scaled to [0;1] range. Then, the accuracy of five classifiers was compared. Leave-one-subject-out validation was used, i.e., the classifiers were trained with 10 subjects and tested on the remaining one. The procedure was repeated 11 times so that all the subjects were used for testing. Finally, the average accuracy was computed. This flow of operations was repeated 6 times, varying the input data: accelerometer and/or gyroscope from dominant hand or both hands.

#### 3.5.2. Binary vs. Multi-Class Classification

Increasing the size of fixed-length windows should increase complex activities recognition performance [12]. However, window size cannot increase indefinitely. Thus, the size of fixed-length windows was set to 10 s, in order to optimize the trade-off between window size and overall recognition performance of this segmentation approach. Notwithstanding, the ability to recognize drinking, an activity which is basically a complex gesture and usually lasts less than 10 s, with such windows was questioned. This brought forward the advantages of creating two different binary classification models, one for each activity of interest, instead of one multi-class model for the simultaneous distinction of eat, drink and other activities, since using several models would enable customization of parameters, such as the segmentation method and the classifier employed. A second experiment with Dataset 1 took place to verify this hypothesis. Since this dataset was composed solely by perfectly segmented activities, it was safe to assume that each computed window corresponded undoubtedly and totally to its assigned label, regardless of the segmentation algorithm employed. The classification performance would, therefore, be associated with how well the segmentation algorithms were able to capture the distinctive characteristics of the activities of interest.

The performance of six classifiers, trained offline with instances of length 33 (number of features that resulted from the process of feature selection), was compared following 4 different approaches associated with different segmentation and labeling methods. First, multi-class labels were assigned and the classifiers were trained with features extracted from 10 s windows with 25% overlap. Then, these were trained with features extracted from dynamic windows, associated to multi-class labels. After that, the feature vectors obtained from 10 s windows were associated with eating vs. non-eating labels, and the ones generated from dynamic windows associated with drinking vs. non-drinking labels. Every approach employed the SMOTE + ENN algorithm for class balancing of the training data. Leave-one-subject-out validation was performed and average accuracy was computed.

#### 3.5.3. Semi-Hierarchical Approach and Branch Optimization

**Figure 2:**
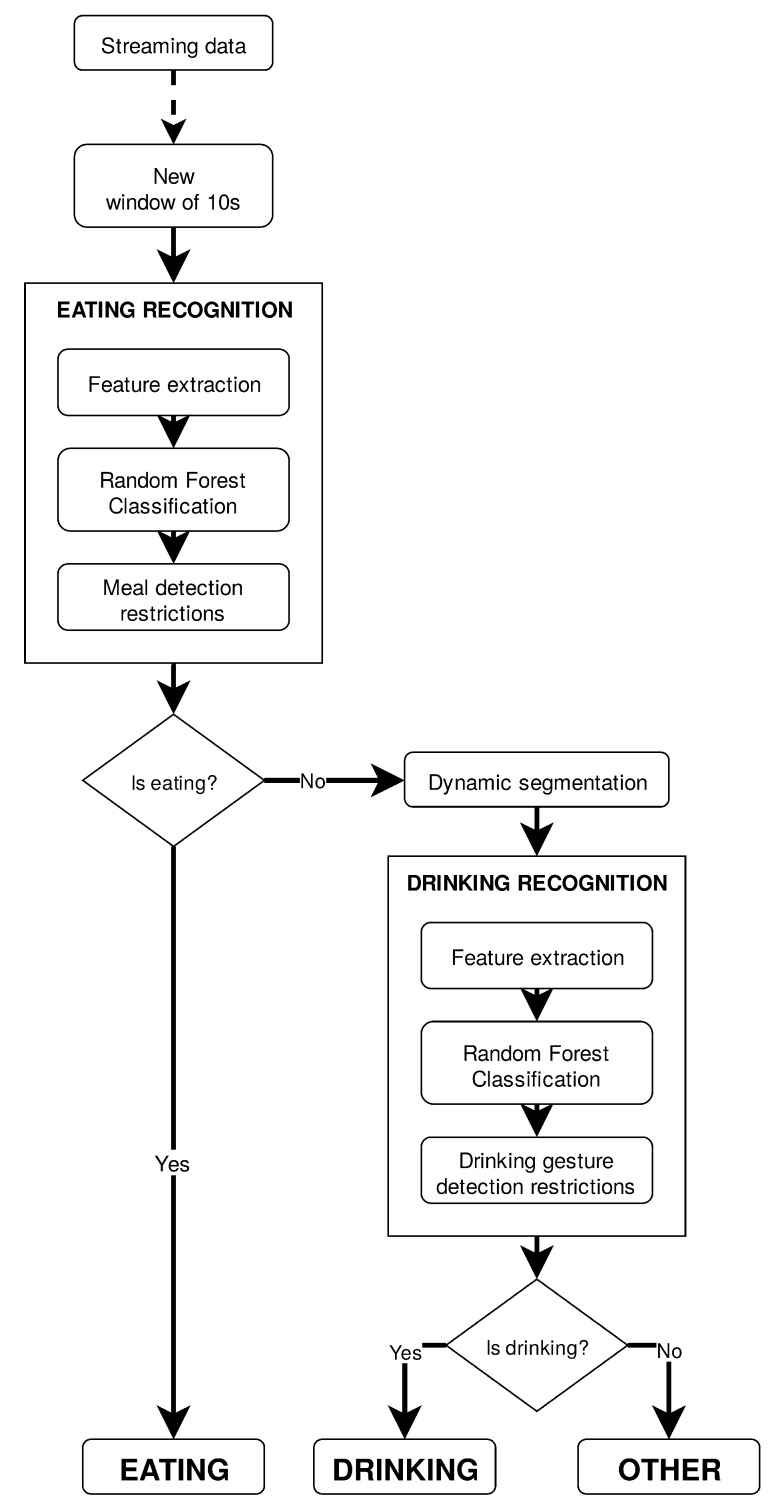
Semi-hierarchical flow of operations of the eating and drinking recognition algorithm. Other includes all non-eating and non-drinking daily living activities.Semi-hierarchical flow of operations of the eating and drinking recognition algorithm.

Adopting a semi-hierarchical approach based on the constitution of two different classification models of binary output, as Figure 2 illustrates, implied the selection of the most adequate classifier to recognize the activities of interest. Concerning eating recognition, this process took place with 3 classifiers on the table—MLP, Random Forest, and also HMM, since its performance might had been impaired by the nature of the previous experiment, i.e., the lack of sequential dependence of data—and by computing another performance metric that not only assessed the representativeness of the model (like accuracy does), but also how discriminative it is. The Area Under the receiving operating characteristic Curve (AUC) is a measure of how above random chance true positive and false positive rates really are. The training set was enlarged to feature not only data from Dataset 1, but also from Dataset 3. Dataset 2 was used as the testing set. Since, during a meal it is common for one to perform random sporadic movements that can be misleading for the classifier, resulting in non-eating classifications among an eating period, classification results were assessed in 1 min periods, i.e., it was only considered an eating moment if over three 10 s-windows were classified as eating in a six-window span (10 s ×6=1 min). In this sense, AUC was computed from the logarithmic probabilities associated with the predictions of each classification model, averaged per 1 min periods. The drinking recognition classifier was selected based on the results from the experiment made clear in Section 3.5.2. Training of the drinking recognition model was supervised and performed offline with data from Dataset 1, every continuous acquisition from Dataset 2 in which drinking events occurred, and drinking data from Dataset 3. Features were associated with drinking vs. non-drinking labels. This selection of datasets was carried in order to take into account class balancing concerns, since the drinking activity was a pronounced minority in the overall acquired set. The training set presented a class distribution of 1 drinking instance to 3.5 non-drinking instances.

**Figure 3:**
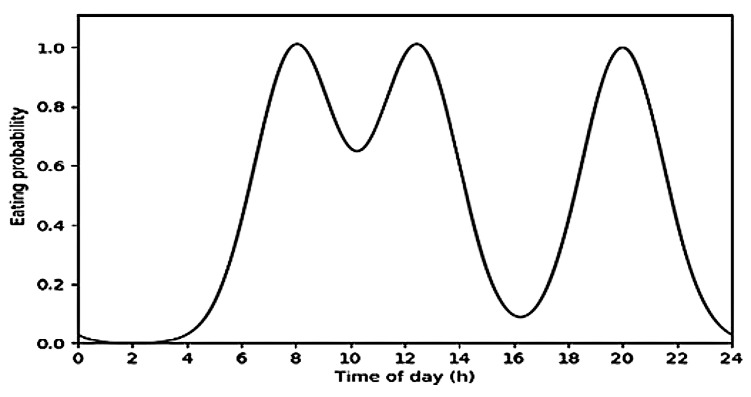
Estimation of the probability of having a meal by time of day.Estimation of the probability of having a meal by time of day.

The scope of this work does not exactly aim the recognition of every single time a user eats something (e.g., taking a piece of chocolate, or having a taste of the food being cooked). It is, however, of extreme importance that every performed meal is detected. Therefore, a set of extra restrictions was brought forward for application on top of the eating classification model output, that should lead to the sole detection of *meaningful* eating moments:

- *Probability of eating depending on the daily hour (E1):* There are some common practices concerning the relation between eating habits and time of day in western culture countries, which make it overall possible to assume that during some specific times of the day the probability of performing a meal increases and during some other times it can be very low. In this sense, the probability that a user is eating by time of day was estimated by means of the sum of three Gaussian distributions (one for each main meal), each one centered at a reasonable time of the day for that meal to happen and with a standard deviation of 1.5 h. Figure 3 illustrates the outcome of this process. Gaussian distributions were centered at 8 a.m., 12:30 p.m. and 8 p.m., corresponding to breakfast, lunch and dinner, respectively. The probability at each time of day for a meal to happen was then used to filter eating predictions by means of Equation (1), where $p_{class}\left(i\right)$ is the probability of the prediction returned by the classifier for the *i*-th 10 s window, $p_{time}\left(i\right)$ is the meal probability estimation by time that corresponds to that same window, and *N* is the number of previous window-associated probabilities considered to smooth the output (in this case, N=3). If $p_{meal}\left(i\right)>0.5$, then an is eating label was assigned.
  (1) $$p_{meal}\left(i\right)=\frac{\sum_{n=0}^{N}p_{class}\left(i-n\right)\times p_{time}\left(i-n\right)}{N}$$
- *Minimum duration of a meal (E2):* Statistics indicate that the OECD country which spends fewer time per day eating (UK) spends 1 h, on average, in this process [36]. This means that, even if four meals take place (breakfast, lunch, afternoon snack, dinner), these would last for 15 min on average, which could support the hypothesis that eating periods that last for less than a 5 to 10 min would not correspond to a meal period and, thus, would not qualify as meaningful in the context of this work. Following these ideas, a 5 min threshold was set, i.e., eating periods shorter than 5 min would not be considered. This threshold is also coherent with the purpose of issuing medication-related reminders, since triggering them 5 min into a meal would optimize the trade-off between time opportunity and guaranteeing that a meal is taking place, in order not to mislead the user.
- *Energy peaks before meals (E3):* The hypothesis of a third restriction was based on the work of [7], in which meals were considered to be preceded and succeeded by energy peaks of the acceleration signal from the wrist of the user. In fact, this assumption is supported by daily evidences. Hand movements are usually slow and small during a meal, but before it takes place it is necessary to prepare it, grab some tools or wash hands, and, after it, several other movements should also occur (e.g., cleaning the dishes). The eating activity would, therefore, happen during a “valley” in the energy of the acceleration signal, preceded and succeeded by energy peaks. In the present case, this concept was used as a preventive measure to avoid false positives, by only considering that a meal is taking place if it was preceded by said peak. Energy computation was implemented as in [7].

Furthermore, each drinking event was found to have at least two rotational magnitude peaks (one when a drinking utensil is grabbed and carried to the mouth, and another when it is put back). Thus, it was expected that the magnitude drop after a peak would often match the end of a dynamic window. Following this consideration, both dynamic segmentation approaches, DW and DFW, were tested, and its impact in drinking recognition performance compared. Since most drinking moments lasted 5–8 s, DFW’s *t* parameter was tuned to 10 s, coinciding with the size of eating recognition fixed-length windows. Moreover, a false positive occurrence preventive measure was put in place: positive drinking predictions were only considered true drinking moments if these featured at least one rotational magnitude peak (D1).

The validation set (excluding the all-day acquisition) was used to assess classification performance and the impact of each proposed post-processing step. Such a test set enabled a proper comparison of approaches since it featured a period during which it was guaranteed that a meal would be performed. In addition, it provided a proper basis on which the meal probability estimation restriction could be tested, since it included a time of maximal eating probability. Three performance metrics suitable for dealing with highly imbalanced classes were computed, taking into account that eating and drinking corresponded to 6.33% and 0.88% of the total time, respectively. Sensitivity and specificity indicate the ratio between true positive and negative predictions and the number of positive and negative instances, respectively. The number of false positives per hour of acquisition (FP/h) was brought as a way to assess the occurrence of false positive predictions. This metric was preferred to precision, since, in this case, the relation between true positives and the total number of positive predictions was not as important as the frequency with which FP occur. These metrics enabled a performance comparison between solely considering the direct classification output and combining it with further processing steps, in both eating and drinking recognition.

#### 3.5.4. Validation

In order to assess the performance of the final algorithm, a two-stage validation approach took place using all data from the validation sets, so that we could assess (1) the algorithm’s suitability for all-day utilization (using data collected over the entire day), and (2) its user-independent nature (namely with data from the target senior population), even at daily times of high meal probability (using data collected around lunch periods). These experiments should provide an adequate testbed for the system, since they should fairly assess the impact of the meal probability estimation restriction, by considering its impact both over a day and around periods prone to the occurrence of meals.

Recognition results were presented in terms of precision, recall and F1-score to enable comparison with related works.

## 4. Results

### 4.1. Sensor Selection

Table 4 exhibits the classification results of the sensor selection experiment. Decision tree, MLP and HMM were found to perform better with data from the dominant wrist of both accelerometer and gyroscope. This fact based the decision to only use data from one device, placed in the dominant wrist of the user, even though inertial data from both wrists seemed to slightly improve *k*-NN and Naive Bayes classification results. The fact that using only one device would reduce the obstructiveness of the system also weighted in the decision.

### 4.2. Binary vs. Multi-Class Classification

**Figure 4:**
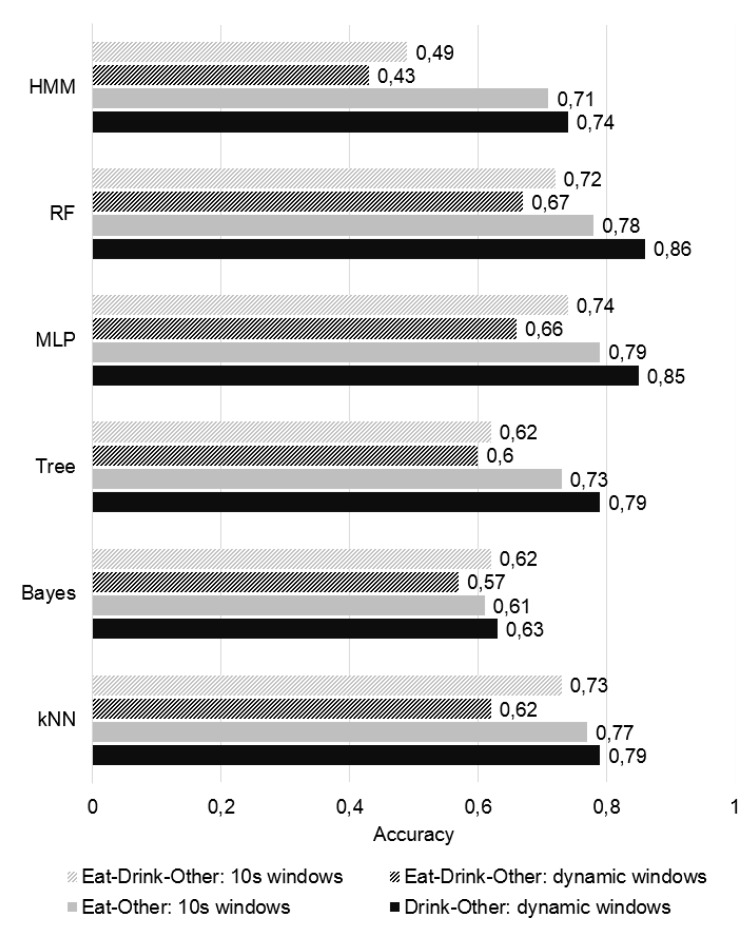
Comparison between overall accuracy of multi-class (striped) vs. binary (full) classification models generated in the sequence of different segmentation methods.Comparison between overall accuracy of binary vs. multi-class classification models generated in the sequence of different segmentation methods.

The assumption that maintaining the same segmentation approach for both activities might impair the recognition was confirmed by the results reported in Figure 4. When assessing multi-class classification, better results were achieved for fixed-length windows, since large windows are frequently associated with a better recognition of complex activities, as previously discussed. Dynamic windows performed poorly in this case, because these over-segment the data. Overall, binary classification was proved to be advantageous in the recognition of both activities. The most significant difference was found for HMM, which greatly improved its classification results with the binary classification approach. Dynamic windows seemed to be a good approach to segment drinking gestures, associated with an accuracy of up to 86% with Random Forest. By being a more complex activity, eating recognition was expected to be more difficult, which is supported by the results. Nevertheless, an accuracy of up to 79% was achieved with MLP.

Out of the 3 approaches which performed eating recognition, fixed-length segmentation of the data stream was associated to better performance in identifying this activity, with MLP and Random Forest classification proving to provide the most accurate results. Concerning drinking classification, the best performance was achieved using the dynamic segmentation algorithm proposed by [11] in combination with a Random Forest model. Following these conclusions, eating and drinking recognition performance were separately studied and improved, aiming a solution in which the activities of interest would be classified by two distinct recognition models, relying in different segmentation approaches.

### 4.3. Semi-Hierarchical Approach and Branch Optimization

Table 5 presents the average AUC for each eating classification model. Random Forest presented significantly better results than MLP and HMM in this experiment. Therefore, it was considered the best classifier for eating recognition. Thus, it was employed in all further experiments, and integrated the final recognition model. The same type of classifier was also selected for the purpose of drinking recognition, as supported by the results presented in Figure 4.

#### Post-Processing Layer Restrictions Assessment

The results reported in Table 6 enable the comparison between approaches which led to branch optimization. Since eating recognition was performed with minute precision, eating-related FP are presented in min. On its hand, concerning drinking recognition, and to minimize the impact of annotation imprecision, it was considered that a drinking event was correctly identified if a positive prediction occurred within 10 s from the ground truth. In this sense, FP correspond to positive predictions that occurred when a true drinking moment did not, in a 10 s time-span.

The metrics extracted from the eating predictions of the Random Forest classifier with minute smoothing indicate that, while almost every eating period was classified as such, many false positive instances occurred. Indeed, during a typical day, there are many activities that might resemble using cutlery, the hand-to-mouth movement and many other hand/wrist movements that are common during a meal. Many of the misclassified instances corresponded to isolated min, usually when the user was performing an activity over a table. The significance of this problem increased with the duration of said activity (e.g., one of the seniors spent approximately an hour playing domino over a table, during which time many misclassified eating predictions were verified). The 5 min threshold restriction had a positive impact in the results, fighting against sporadic occurrences of FP. The meal probability estimation proved to be the single restriction with better impact in recognition performance, which shall be even more notorious in all-day applications. On the other hand, the hypothesis of [7] proved not to suit this application, since many eating periods were not preceded by an energy peak in the acceleration signal, probably because of the conditions of some acquisitions (e.g., the waiting period at the table before lunch for the seniors did not produce an energy peak before the meal started). While it is an approach worth of further investigation, it was important to realize that not all eating periods verify this premise, since it is always dependent on the before-meal context. The approach which led to better results consisted in combining the 5 min threshold restriction with the estimation of meal probability, which minimized the occurrence of FP to ≈4 misclassified min per hour while maintaining a good TP classification rate (all meals were identified, even though some of its min were not, usually in the beginning and ending of the meal, which may be associated with the labeling problem, discussed in the previous section). Thus, this was the eating recognition approach finally selected.

It is also possible to assess the positive impact of restraining the dynamic segmentation of data to 10 s-windows instead of unrestrictedly to the data stream in terms of minimizing the occurrence of false negatives. However, it increases overall sensitivity at the expense of decreasing specificity. This problem was compensated by the rotational magnitude peak restriction, which minimized the occurrence of FP to 5 misclassified drinking instances per hour. This test set also revealed that there were some unforeseen activities frequently misclassified as drinking, e.g., bitting nails. In fact, one of the users did this frequently, and those misclassified instances corresponded to ≈64% of the total number of FP. The introduction of that activity in the training set constitutes, therefore, a future step that shall improve drinking identification in free-living conditions.

All in all, these results verified that filtering the classification output based on what is known about the activities of interest is a very good approach to improve overall recognition performance with very simple and computationally efficient strategies.

### 4.4. Validation

The algorithm’s capabilities for all-day utilization were assessed using the all-day test set. The results presented in Table 7 indicate the performance of the algorithm concerning sole eating/drinking recognition and the simultaneous recognition of both activities. All meals were detected, even though some of their initial and final min were not. Once again, the problem of labeling data is clear, and a more accurate method might be in demand to further assess the potential of activity recognition algorithms, in spite of the advantages in terms of privacy of manually logging activities. Even though we were considering a period of 16 h, out of which only a small percentage was spent eating, the eating-class precision value indicates a fair decrease of FP occurrences per hour, when compared to eating recognition results reported in Table 6, reinforcing the efficacy of the meal probability estimation restriction—only one eating period was mistakenly predicted. Moreover, FP predictions could only occur in periods when the meal probability estimation is larger than 0.5, which minimizes their negative impact by limiting them to an opportune time-span. The potential of such an approach can even increase by incrementally adapting the center of the Gaussian distribution for each meal to the user’s habits, leading to better user-dependent results. Drinking recognition performance results for this 16 h acquisition indicate that the algorithm performs well in detecting only true drinking moments even when unrestricted, natural and spontaneous movements are performed. Nonetheless, to enrich the classification model with data from more activities that might resemble drinking, such as bitting nails, but are not, might be an adequate next step to improve its performance. Overall, the entire algorithm identified both activities of interest with a weighted F1-score of 97%, which was considered very promising towards the success of the tool.

The entire validation set was finally taken to assess the global performance of the algorithm (Table 8), including not only all-day data but also 15 other hours of data from 4 different users acquired at daily times of high meal probability. This validation was particularly demanding in terms of eating recognition, due to the meal probability estimation restriction. Indeed, 48% of the 31 h of data corresponded to daily periods of over 70% probability of performing a meal. Even so, less than 2 h of data were mistakenly classified as eating, an evidence of the decent performance achieved even at the most critical times. This fact justifies the modest value of unweighted eating recognition precision. Drinking recognition results were mostly coherent with those reported in Table 6 and Table 7. Due to class imbalance, weighted metrics are the ones which better describe overall performance, delivering an F1-score of 93%. This value is particularly suggestive of the good performance of the algorithm under the demanding circumstances under which these experiments took place.

## 5. Discussion of Results

The results of these experiments base the main contributions of this work, and are worthy of a thorough discussion so that its pertinence is clear.

The in-the-wild validation tests unveiled the challenges of designing training sets to base predictions in free-living conditions. Besides providing data for the training stage, these sets of data also enabled sensor selection: both accelerometer and gyroscope were found to hold important eating and drinking distinctive information, even when considering solely the dominant hand of the users. The training sets were planned to feature the most distinct daily activities and specifically intended to capture the diversity of movements inherent to free-living. However, some unseen activities were still performed in the in-the-wild validation, unveiling handicaps of the classification models, namely misclassification of bitting nails as drinking, and of performing small movements at a table (e.g., playing cards) as eating. The training set could therefore be improved, as it was possible to survey new activities that should be considered in the training process. Despite that, the collected set provided an adequate basis for a robust classification, as the models’ handicaps were partially compensated by post-processing the classification output (Table 6).

Verifying the improvement of performance associated to binary classification as opposed to multi-label classification inspired the implementation of a semi-hierarchical eating and drinking recognition approach. The potential of hierarchical approaches has been explored in related works [1,12], associated to results that should motivate further research from the daily living activity recognition community. One of the main advantages of such a technique is the possibility of customization of branches. In this case, it enabled the application of different data stream segmentation techniques, and a sequence of post-processing restrictions taking advantage of the characteristics of each activity of interest. These restrictions proved to have a significant positive impact in the recognition performance, supporting the work of [12], which drew attention to the importance of processing the classification output to improve the recognition of complex activities. As such, these conclusions should contribute towards more mature eating and drinking recognition algorithms, as the proposed post-processing techniques can be further combined with other approaches. Our results also verify an increase of the sensitivity of drinking recognition when using DFW instead of the typical DW segmentation. This outcome should bring awareness for the potential of the method in the detection of specific anomalies, like the drinking movement, in data streams, without impairing other running classification mechanisms.

Comparing this work with others that preceded it is not a straightforward task. Firstly, because of its very own purpose of simultaneous eating and drinking recognition (and distinction) in free-living conditions, which, to the best of the authors’ knowledge, is a novel intention. Moreover, this work aims the issuing of reminders in opportune daily moments and not dietary monitoring or food journaling, which is the main goal of the prior art, and has an impact in the definition of prerequisites for the system. Nonetheless, several techniques used in related works were combined and tested [5,7,11,12,13], while being framed within the purpose of our work, providing a starting point for creating a more mature solution which built upon the prior art. Considering eating recognition performance of the works that cared to preserve some of our important prerequisites, namely free-living utilization (and, thus, validated in free-living conditions) by using inertial data from an unobtrusive wristband, the results reported in this work appear to outperform those reported by [7] and [6], by achieving 97% F1-score for eating recognition in the all-day test set and 93% in the entire validation set with 5 different users, which included data from senior volunteers. Moreover, [7] proposed an algorithm of offline response and [6] an approach that looks for eating moments every hour, which would not suit our goal of real-time eating detection with, at least, minute precision to enable the reminders. On the other hand, both [7] and [6] validated their approaches in larger datasets, meaning that further data collections might be in demand for a fairer comparison of approaches. In [8] and [9], eating periods corresponded to 36% and 11%, respectively, of the total time of the free-living datasets that were used, hindering comparison with our system. Those works also involved more obstructive systems and algorithms of, apparently, higher computational cost that this one, comprising a large number of features, some of which from the frequency domain. No works of drinking recognition in free-living conditions were found for comparison. The conditions in which our study took place were also much more challenging than those of [10], since that was an in-the-lab study, a controlled environment which did not account for all the challenges of free-living sporadic activity recognition. Therefore, slightly worse results were expected in this case. Nevertheless, most drinking events were correctly identified for all users of the test sets and activities associated with FP were surveyed, in order to improve the next iteration of our drinking classification model.

This study presented, however, some limitations. On one hand, the fact that it was only possible to perform an all-day acquisition of data from 1 young volunteer. Since it is intended that users use this system throughout the entire day, all-day data are the most useful to reveal potential handicaps of the system. Thus, having only one test subject in that test set is a clear limitation of the validation process, which hinders the comparison with previous works. This fact was compensated by considering the data of 4 other subjects, including 2 seniors, in the validation process, even though it was not possible to perform all-day acquisitions with them. The system was also not tested with left-handed users, so its impact in the results is yet to know, despite the inclusion of data from a left-handed subject in the training set. Analogously, we were not able to study the generalization of our models for different age groups, even though our tests with data from young and older adults did not evidence a particular drop of performance for any specific group. In addition, in theory, age should not particularly impact the outcome of the post-processing layer of restrictions. Furthermore, this system depends on inertial data from the dominant wrist of users, which means that movements performed with the non-dominant hand are lost. While the impact of this problem is dimmed for the eating activity, since it usually involves the use of both hands, it means that drinking events are completely lost if performed with the non-dominant hand, a problem that must be revisited in further research. The final recognition algorithm also presents some limitations. The 5-min restriction, which increases confidence in eating predictions, is one of them: while the algorithm detects eating with minute precision, it requires a 5-min confidence period to pass before it decides on a true eating moment. While this is not very important considering the goal of issuing reminders, it leaves room for improvement. As previously discussed, it is also still difficult for the classification models to distinguish some daily activities from eating and drinking, meaning that further data collections of some specific activities might be in demand to improve the training set.

## 6. Conclusions

This work proposed a new algorithm of activity recognition, which built upon the prior art to deliver a user-independent solution relying on a sensing system of low obstructiveness for the simultaneous recognition of eating and drinking activities in free-living conditions. An F1-score of 97% was attained for the recognition of the activities of interest in an all-day acquisition from one unseen user. The validation was also extended to feature data from 4 extra subjects, 2 of which were older adults, testing the user-independent nature of the algorithm, and achieved an F1-score of 93%. As such, the system shall provide an adequate basis for triggering useful smart reminders for autonomously living seniors, since it delivered promising results while being tested under user-independent and free-living conditions, important objectives of this work. Moreover, the system relied on simple wrist sensorization, which shall promote user acceptance by an older population.

As future work, we intend to enrich the classification models with data from activities that were frequently mistaken with the activities of interest (e.g., in case of eating, activities that involve small movements over a table, like playing board games; in case of drinking, bitting nails). Improving the training set shall also lead to general optimizations of the algorithm, e.g., reducing the number of features and/or trees of the Random Forest classifiers, taking advantage of the possibility of customizing each branch of the algorithm, namely the recognition models. The solution shall also be mobile implemented and tested in real-time.

## Figures and Tables

**Table 1:** Supervised Human Activity Recognition (HAR) works that recognize the eating activity among other Activities of Daily Living (ADL).

Work	Summary of Proceedings	
[15]	Recognized activities	Walking, walking carrying items, sitting & relaxing, working on computer, standing still, eating or drinking, watching TV, reading, running, bicycling, stretching, strength-training, scrubbing, vacuuming, folding laundry, lying down & relaxing, brushing teeth, climbing stairs, riding elevator and riding escalator
	Sensors	Accelerometers
	Features	Mean, energy, frequency-domain entropy, and correlation
	Classifiers	Decision tree C4.5
	Eating recognition metrics	Accuracy: 89%
[16]	Recognized activities	Sitting, sit-to-stand, stand-to-sit, standing, walking, typing on keyboard, using the mouse, flipping a page, cooking, eating
	Sensors	Accelerometers and location tracker
	Features	Mean and variance of the 3D acceleration
	Classifiers	Dynamic Bayesian Network
	Eating recognition metrics	Accuracy: 80%
[17]	Recognized activities	Brushing teeth, dressing/undressing, eating, sweeping, sleeping, ironing, walking, washing dishes, watching TV
	Sensors	Accelerometer, thermometer and altimeter
	Features	Mean, minimum, maximum, standard deviation, variance, range, root-mean-square, correlation, difference, main axis, spectral energy, spectral entropy, key coefficient
	Classifiers	Support Vector Machines
	Eating recognition metrics	Accuracy: 93%
[12]	Recognized activities	Standing, jogging, sitting, biking, writing, walking, walking upstairs, walking downstairs, drinking coffee, talking, smoking, eating
	Sensors	Accelerometer, gyroscope and linear acceleration sensor
	Features	Mean, standard deviation, minimum, maximum, semi-quantile, median, sum of the first ten FFT coefficients
	Classifiers	Naive Bayes, *k*-Nearest Neighbors, Decision Tree
	Eating recognition metrics	F1-score: up to 87%

**Table 2:** Summary of eating detection and recognition works, validated in free-living conditions.

	Sensor Type	No. Sensors	Features	Classifier	Performance Metrics
[7]	Inertial	1	Manipulation, linear acceleration, amount of wrist roll motion, regularity of wrist roll motion	Naive Bayes	Accuracy (weighted): 81%
[8]	Multimodal	2	MAV, RMS, maximum, median, entropy, zero crossings, no. peaks, average range, wavelength, no. slope sign changes, energy of the frequency spectrum, energy spectrum, entropy spectrum, fractal dimension, peak frequency, standard deviation, and other features derived from the aforementioned	ANN	Accuracy: 90%
[6]	Inertial	1	Mean, Variance, Skewness, Kurtosis, RMS	Random Forest	F1-scores: 71–76%
[9]	Inertial	2	RMS, variance, entropy, peak power, power spectral density, zero crossing, variance of zero crossing, peak frequency, number of auto-correlation peaks, prominent peaks, weak peaks, maximum auto-correlation value, first peak	Random Forest	Accuracy: 93%; F1-score: 80%

**Table 3:** Description of the datasets.

**DATASET 1**				
**Activity**	**Description**	**No. Subjects**	**Planned Time/Subject (s)**	**Total Acquisition Time (s)**
Eat	With spoon With fork and knife With hands, sitting With hands, standing	9 11 11 11	120 120 60 60	4055
Drink	Grab mug, sip, put mug back Get water bottle from a bag, long sip, put it back Grab cup, sip, put cup back Drink while standing	11 9 11 9	90 90 90 90	3727
Other	Make a phone call Comb hair Walk Scratch face Working with keyboard and mouse Texting Writing with a pen	11 10 11 11 11 11 11	60 60 60 30 60 30 60	4114
**DATASET 2**				
**ID**	**Sequence of Activities**	**Approx. Total Duration (s)**	**Approx. Eating Duration (s)**	**Approx. Drinking Duration (s)**
1	Walk → Prepare meal → Have breakfast → Drink tea while watching TV → Brush teeth → Comb hair → Apply facial cream	1609	288	175 (20 events)
2	Walk → Have dinner → Drink from a cup → Have a conversation → Make a phone call → Play with smartphone	2425	1748	13 (2 events)
3	Eat dessert with a spoon → Descend stairs → Walk → Drink water from a bottle → Walk → Ascend stairs → Brush teeth → Comb hair	521	398	7 (1 event)
4	Descend stairs → Walk → Play table football → Stand in a line → Walk with a tray → Have lunch → Walk	3859	1038	0
5	Wash dishes → Drink water from a bottle → Prepare a meal → Have lunch → Work with mouse and keyboard → Drink tea from a mug while working with a computer	2620	608	105 (14 events)
**DATASET 3**				
**Activity**	**Description**	**No. Seniors**	**Total Acquisition Time (s)**	
Eat	Having lunch Having an afternoon snack	6 2	9278 666	
Drink	Simulate grabbing a cup/bottle, sipping and putting the object back	16	236	

**Table 4:** Accuracy of the classification using accelerometer (A) and/or gyroscope (G) data from the dominant (D) vs. both (B) wrists.

Wrists		Sensors		Classifiers *				
D	B	A	G	kNN	NB	DT	MLP	HMM
X		X		0.65	0.64	0.56	0.66	0.73
X			X	0.56	0.48	0.47	0.56	0.59
X		X	X	0.72	0.66	**0.61**	**0.75**	**0.75**
	X	X		0.70	**0.68**	0.58	0.68	0.69
	X		X	0.62	0.51	0.46	0.64	0.59
	X	X	X	**0.75**	0.64	0.57	0.74	0.73

* Bold values highlight highest achieved accuracy for each classifier.

**Table 5:** Average Area Under the receiving operating characteristic Curve (AUC) for each eating classification model.

	MLP	HMM	Random Forest
AUC	0.77 (+/− 0.21)	0.75 (+/− 0.22)	0.84 (+/− 0.12)

**Table 6:** Performance metrics of eating and drinking recognition, based on Random Forest classification, with respect to the added restrictions.

Activity	Segmentation + Post-Processing	Sensitivity	Specificity	FP/h
Eat	FW FW + E1 FW + E2 FW + E3 FW + E1 + E2 *	0.95 0.84 0.93 0.23 **0.73**	0.42 0.81 0.56 0.81 **0.93**	34.47 11.13 25.80 10.93 **4.27**
Drink	DW DFW DFW + D1 *	0.57 0.63 **0.63**	0.99 0.98 **0.99**	5.33 6.13 **5.06**

* Best segmentation/post-processing combination for eating and drinking detection.

**Table 7:** All day acquisition recognition performance.

EATING *							
**Confusion Matrix**					**Performance Metrics**		
	Eat	Not eat	**Total**		Precision	Recall	F1-score
Eat Not eat	25 14	11 904	**36** **933**		0.64 0.99	0.69 0.98	0.67 0.99
**Total/Avg**	**39**	**915**	**969**		**0.97**	**0.97**	**0.97**
DRINKING *							
**Confusion Matrix**					**Performance Metrics**		
	Drink	Not drink	**Total**		Precision	Recall	F1-score
Drink Not drink	26 10	14 5689	**40** **5699**		0.72 1.00	0.65 1.00	0.68 1.00
**Total/Avg**	**36**	**5703**	**5739**		**1.00**	**1.00**	**1.00**
OVERALL *							
**Confusion Matrix**					**Performance Metrics**		
	Eat	Drink	Other	**Total**	Precision	Recall	F1-score
Eat Drink Other	150 5 79	0 24 10	66 11 5380	**216** **40** **5469**	0.64 0.71 0.99	0.69 0.60 0.98	0.67 0.65 0.98
**Total/Avg**	**234**	**34**	**5457**	**5725**	**0.97**	**0.97**	**0.97**

* Total no. samples per class and average of performance metrics are highlighted in bold. Rows and columns correspond to true and predicted labels, respectively.

**Table 8:** Complete validation set recognition performance.

	Precision	Recall	F1-Score	No. Samples
Eat	0.39	0.77	0.52	552
Drink	0.37	0.62	0.46	81
Other	0.99	0.93	0.96	10737
**Total/Avg**	**0.95**	**0.92**	**0.93**	**11370**

## References

[1] Anderez D.O., Appiah K., Lotfi A., Langesiepen C. A hierarchical approach towards activity recognition. Proceedings of the 10th International Conference on Pervasive Technologies Related to Assistive Environments. ACM.

[2] O’brien T., Troutman-Jordan M., Hathaway D., Armstrong S., Moore M. (2015). Acceptability of wristband activity trackers among community dwelling older adults. Geriatr. Nurs..

[3] Holzinger A., Searle G., Prückner S., Steinbach-Nordmann S., Kleinberger T., Hirt E., Temnitzer J. Perceived usefulness among elderly people: Experiences and lessons learned during the evaluation of a wrist device. Proceedings of the 2010 4th International Conference on Pervasive Computing Technologies for Healthcare (PervasiveHealth). IEEE.

[4] Rasche P., Wille M., Theis S., Schäefer K., Schlick C.M., Mertens A. Activity tracker and elderly. Proceedings of the 2015 IEEE International Conference on Computer and Information Technology; Ubiquitous Computing and Communications; Dependable, Autonomic and Secure Computing; Pervasive Intelligence and Computing (CIT/IUCC/DASC/PICOM).

[5] Figo D., Diniz P.C., Ferreira D.R., Cardoso J.M. (2010). Preprocessing techniques for context recognition from accelerometer data. Pers. Ubiquitous Comput..

[6] Thomaz E., Essa I., Abowd G.D. A practical approach for recognizing eating moments with wrist-mounted inertial sensing. Proceedings of the 2015 ACM International Joint Conference on Pervasive and Ubiquitous Computing. ACM.

[7] Dong Y., Scisco J., Wilson M., Muth E., Hoover A. (2014). Detecting periods of eating during free-living by tracking wrist motion. IEEE J. Biomed. Health Inform..

[8] Fontana J.M., Farooq M., Sazonov E. (2014). Automatic ingestion monitor: A novel wearable device for monitoring of ingestive behavior. IEEE Trans. Biomed. Eng..

[9] Bedri A., Li R., Haynes M., Kosaraju R.P., Grover I., Prioleau T., Beh M.Y., Goel M., Starner T., Abowd G. (2017). EarBit: Using wearable sensors to detect eating episodes in unconstrained environments. Proc. ACM Interact. Mob. Wearable Ubiquitous Technol..

[10] Amft O., Bannach D., Pirkl G., Kreil M., Lukowicz P. Towards wearable sensing-based assessment of fluid intake. Proceedings of the PerCom Workshops.

[11] Kozina S., Lustrek M., Gams M. Dynamic signal segmentation for activity recognition. Proceedings of the International Joint Conference on Artificial Intelligence.

[12] Shoaib M., Bosch S., Incel O.D., Scholten H., Havinga P.J. (2016). Complex human activity recognition using smartphone and wrist-worn motion sensors. Sensors.

[13] Ramos-Garcia R.I., Muth E.R., Gowdy J.N., Hoover A.W. (2015). Improving the recognition of eating gestures using intergesture sequential dependencies. IEEE J. Biomed. Health Inform..

[14] Cardinaux F., Bhowmik D., Abhayaratne C., Hawley M.S. (2011). Video based technology for ambient assisted living: A review of the literature. J. Ambient Intell. Smart Environ..

[15] Bao L., Intille S.S. (2004). Activity recognition from user-annotated acceleration data. Proceedings of International Conference on Pervasive Computing.

[16] Zhu C. (2011). Hand Gesture and Activity Recognition in Assisted Living through Wearable Sensing and Computing. Ph.D. Thesis.

[17] Chernbumroong S., Cang S., Atkins A., Yu H. (2013). Elderly activities recognition and classification for applications in assisted living. Expert Syst. Appl..

[18] Varkey J.P., Pompili D., Walls T.A. (2012). Human motion recognition using a wireless sensor-based wearable system. Pers. Ubiquitous Comput..

[19] Thomaz E. (2016). Automatic Eating Detection in Real-World Settings with Commodity Sensing. Ph.D. Thesis.

[20] Amft O., Junker H., Troster G. Detection of eating and drinking arm gestures using inertial body-worn sensors. Proceedings of the 9th IEEE International Symposium on Wearable Computers (ISWC’05).

[21] Amft O., Tröster G. (2008). Recognition of dietary activity events using on-body sensors. Artif. Intell. Med..

[22] Junker H., Amft O., Lukowicz P., Tröster G. (2008). Gesture spotting with body-worn inertial sensors to detect user activities. Pattern Recognit..

[23] Lukowicz P., Ward J.A., Junker H., Stäger M., Tröster G., Atrash A., Starner T. (2004). Recognizing workshop activity using body worn microphones and accelerometers. Proceedings of the International Conference on Pervasive Computing.

[24] Lee C., Xu Y. Online, interactive learning of gestures for human/robot interfaces. Proceedings of the IEEE International Conference on Robotics and Automation.

[25] Gomes D., Sousa I. (2019). Real-Time drink trigger detection in free-living conditions using inertial sensors. Sensors.

[26] Pedregosa F., Varoquaux G., Gramfort A., Michel V., Thirion B., Grisel O., Blondel M., Prettenhofer P., Weiss R., Dubourg V. (2011). Scikit-learn: Machine learning in Python. J. Mach. Learn. Res..

[27] Fraunhofer Portugal AICOS (2016). A Day with Pandlets.

[28] Charmant J. (2012). Kinovea.

[29] Shen Y., Muth E., Hoover A. Recognizing eating gestures using context dependent hidden Markov models. Proceedings of the IEEE First International Conference on Connected Health: Applications, Systems and Engineering Technologies (CHASE).

[30] Hanai Y., Nishimura J., Kuroda T. Haar-like filtering for human activity recognition using 3d accelerometer. Proceedings of the IEEE 13th Digital Signal Processing Workshop and 5th IEEE Signal Processing Education Workshop.

[31] Breiman L., Friedman J., Olshen R., Stone C. (1984). Classification and Regression Trees.

[32] Chawla N.V., Bowyer K.W., Hall L.O., Kegelmeyer W.P. (2002). SMOTE: Synthetic minority over-sampling technique. J. Artif. Intell. Res..

[33] Wilson D.L. (1972). Asymptotic properties of nearest neighbor rules using edited data. IEEE Trans. Syst. Man Cybern..

[34] Batista G.E., Prati R.C., Monard M.C. (2005). Balancing strategies and class overlapping. Proceedings of International Symposium on Intelligent Data Analysis.

[35] More A. (2016). Survey of resampling techniques for improving classification performance in unbalanced datasets. arXiv.

[36] Statista T.S.P. Time Spent Eating and Drinking by Men and Women in OECD Countries 2016|Statistic, 2016. https://www.statista.com/statistics/521972/time-spent-eating-drinking-countries/.

